# A DAT1 gene and APOE ε4 interaction is associated with apathy and structural brain changes in Alzheimer’s Disease

**DOI:** 10.1002/alz.088450

**Published:** 2025-01-09

**Authors:** Rubina Malik, Derek Beaton, Andrew J. Saykin, Kwangsik Nho, Elizabeth Finger

**Affiliations:** ^1^ Western University, London, ON Canada; ^2^ Data Science & Advanced Analytics, St. Michael’s Hospital, Unity Health Toronto, Toronto, ON Canada; ^3^ Indiana Alzheimer's Disease Research Center, Indianapolis, IN USA; ^4^ Indiana Alzheimer’s Disease Research Center, Indiana University School of Medicine, Indianapolis, IN USA; ^5^ Indiana University School of Medicine, Indianapolis, IN USA; ^6^ Indiana Alzheimer Disease Research Center, Indianapolis, IN USA; ^7^ Department of Clinical Neurological Sciences, University of Western Ontario, London, ON Canada

## Abstract

**Background:**

Apathy in patients with Alzheimer’s disease (AD) is associated with significant morbidity. We examined whether interactions between genetic variants related to neurotransmitter systems and regional brain atrophy are associated with apathy in patients with mild cognitive impairment (MCI) and AD.

**Method:**

For 1162 participants in the Alzheimer’s Disease Neuroimaging Initiative, including those with AD, MCI and cognitively normal individuals, a partial least squares correspondence analysis (PLS‐CA) modeled interactions between single nucleotide polymorphisms (SNPs), structural whole‐brain imaging variables, and apathy.

**Result:**

An interaction between apathy, the possession of an APOE (apolipoprotein E) ε4 allele combined with minor homozygosity for the DAT1 (dopamine transporter 1) gene, and brain atrophy.

**Conclusion:**

The results point to an association of a dopaminergic genetic marker and apathy in AD and may inform future design of clinical trials of apathy, as well as new treatment targets.

